# Integration of peripheral blood-based systemic inflammatory indices and retinal imaging using interpretable machine learning for predicting anti-VEGF treatment response in macular edema secondary to retinal vein occlusion

**DOI:** 10.3389/fcell.2025.1732963

**Published:** 2025-12-29

**Authors:** Jiajun Li, Yao Lu, Qianzi Jin, Siqi Wu, Xiangzhong Xu, Qin Jiang, Keran Li

**Affiliations:** 1 Department of Ophthalmology, The Affiliated Eye Hospital of Nanjing Medical University, Nanjing, China; 2 The Fourth School of Clinical Medicine, Nanjing Medical University, Nanjing, China

**Keywords:** anti-VEGF therapy, interpretable machine learning, macular edema, retinal vein occlusion, shapley additive explanations, systemic inflammation indices

## Abstract

**Purpose:**

Macular edema secondary to retinal vein occlusion (RVO-ME) demonstrates considerable inter-individual variability in response to anti-VEGF therapy. While current research has predominantly focused on ocular imaging features and intraocular cytokine profiles, the role of systemic inflammation remains underexplored. This study proposes an interpretable machine learning (ML) framework that integrates peripheral blood-based systemic inflammatory indices with retinal imaging data to predict anatomical outcomes following anti-VEGF treatment in RVO-ME, and to elucidate underlying systemic inflammation–retinal structure interactions.

**Methods:**

This single-center retrospective study included 202 RVO-ME patients receiving a standardized three-injection anti-VEGF regimen. Clinical data, retinal imaging parameters, peripheral blood cell counts, and derived systemic inflammatory indices were collected. Feature selection used least absolute shrinkage and selection operator (LASSO) and Boruta algorithms. Nine ML models were developed and optimized through Bayesian hyperparameter tuning with five-fold cross-validation for model selection, followed by independent test set validation. SHapley Additive exPlanations (SHAP) and Generalized Additive Models (GAMs) provided interpretation and mechanism exploration. A web-based risk calculator was deployed for clinical translation.

**Results:**

Central macular thickness before the third injection (CMT-2), minimum neutrophil-to-lymphocyte ratio (NLR-min), and minimum systemic immune-inflammation index (SII-min) emerged as key predictors. The Random Forest model performed optimally. SHAP and GAMs revealed that exacerbated systemic inflammation (SII-min and NLR-min) attenuated retinal structural treatment benefit (CMT-2), while concurrent elevations in inflammatory and structural burden markedly increased non-response risk. Counterfactual simulation suggested therapeutic gains from targeting systemic inflammation. The calculator based on the optimal model offers visual decision support for early non-responder identification.

**Conclusion:**

This study identifies systemic inflammation–retinal structure synergy as a key mechanism underlying anti-VEGF treatment heterogeneity in RVO-ME, highlights systemic inflammation as a modifiable therapeutic target, and supports personalized treatment strategies to improve clinical outcomes.

## 1 Introduction

Retinal vein occlusion (RVO), the second leading cause of retinal vascular blindness worldwide after diabetic retinopathy (DR), is typically categorized into central retinal vein occlusion (CRVO) and branch retinal vein occlusion (BRVO) (Song et al., 2019; Scott et al., 2020). The most vision-threatening complication of RVO is macular edema (ME), which results from a multifactorial pathophysiological process involving increased retinal venous pressure, impaired capillary perfusion, breakdown of the blood-retinal barrier (BRB), and inflammatory cytokine-mediated vascular leakage (Daruich et al., 2018; Noma et al., 2019; Haydinger et al., 2023). Intravitreal injection of anti-vascular endothelial growth factor (anti-VEGF) agents is currently the first-line treatment for ME secondary to retinal vein occlusion (RVO-ME), with the “3+PRN” regimen widely adopted in clinical practice (Larsen et al., 2016; Campochiaro and Akhlaq, 2021), whereby three consecutive monthly injections are followed by as-needed maintenance according to the disease condition. While the majority of patients demonstrate anatomical and functional visual improvement, approximately 30%–40% exhibit persistent or early recurrent edema after the initial loading phase (Menke et al., 2016; Johari et al., 2024), highlighting substantial inter-individual variability in treatment response and potential biological resistance to anti-VEGF therapy. Consequently, the early identification of poor responders remains a pressing clinical need to guide individualized treatment strategies and improve long-term outcomes.

Noninvasive imaging modalities, such as optical coherence tomography (OCT) and OCT angiography (OCTA), provide a range of quantitative biomarkers—including central macular thickness (CMT), total macular volume, subretinal fluid height, hyperreflective foci, and vascular density—that are increasingly utilized in the clinical evaluation of RVO-ME (Segal et al., 2021; Śpiewak et al., 2023; Karataş et al., 2025). Among these, CMT is the most widely utilized parameter due to its high reproducibility and accessibility in clinical settings, serving as an objective surrogate for both disease severity and therapeutic response. However, accumulating evidence suggests that reliance solely on CMT values or their early changes may be inadequate for predicting long-term treatment outcomes (Bressler et al., 2019; Nagasato et al., 2024). Previous studies have demonstrated that elevated intraocular cytokines such as interleukin-6 (IL-6) and tumor necrosis factor-α (TNF-α) are strongly correlated with the severity of RVO-ME and resistance to anti-VEGF therapy (Wang et al., 2022). However, the clinical applicability of these local biomarkers is significantly constrained: intraocular fluid sampling requires invasive procedures such as anterior chamber paracentesis, which entail risks of complications including endophthalmitis and cataract. Furthermore, high procedural costs and reliance on specialized equipment further restrict their feasibility for routine or longitudinal monitoring. Therefore, there is an urgent need for non-invasive and easily accessible biomarkers that can dynamically capture the inflammatory status in patients with RVO-ME. Notably, individuals with RVO-ME frequently exhibit varying degrees of systemic inflammation, which may contribute to disease progression by altering vascular permeability and facilitating immune cell adhesion and extravasation (Noma et al., 2022). Several systemic inflammatory indices derived from peripheral blood counts, such as the neutrophil-to-lymphocyte ratio (NLR), platelet-to-lymphocyte ratio (PLR), lymphocyte-to-monocyte ratio (LMR), systemic inflammation response index (SIRI), aggregate index of systemic inflammation (AISI), and systemic immune-inflammation index (SII), have demonstrated predictive value in various retinal diseases, including DR and age-related macular degeneration (AMD), largely due to their simplicity, cost-effectiveness, and biological stability (Wang et al., 2023; Gunay, 2024). Nevertheless, comparative evidence regarding their predictive performance in the anatomical response of RVO-ME to anti-VEGF therapy remains limited, and integrated analyses combining these systemic inflammatory indices with ocular imaging parameters are still insufficiently explored.

In recent years, machine learning (ML) has gained increasing attention on the development of medical prediction models due to its ability to process high-dimensional, nonlinear data (Basu et al., 2020; Li et al., 2024). Compared with traditional statistical methods, ML offers superior performance in capturing complex interactions and enhancing predictive accuracy. However, its inherent “black box” nature has hindered clinical adoption. SHapley Additive exPlanations (SHAP), as a state-of-the-art interpretability technique, provides a quantitative framework for attributing the contribution of each input variable to individual predictions, thereby improving model transparency and clinical trustworthiness (Xu et al., 2024; Qi et al., 2025). Previous studies have explored ML-based prediction of treatment outcomes in patients with RVO-ME. For instance, Liang et al. developed an XGBoost model that incorporated clinical and imaging parameters—such as baseline visual acuity, systolic blood pressure, age, and retinal inner layer dysfunction—to predict visual outcomes in RVO-ME patients (Liang et al., 2025). However, the predictive variables in that study were restricted to structural and basic physiological parameters, excluding systemic inflammatory indices—an important yet underexplored dimension of disease pathophysiology. This omission may have constrained the model’s ability to comprehensively capture inter-individual heterogeneity in treatment response.

**FIGURE 1:**
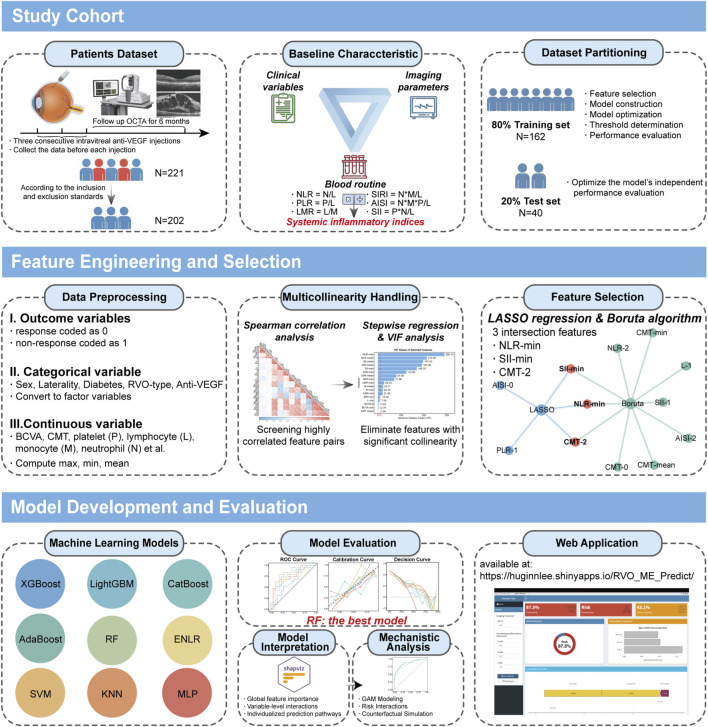
Overview of the ML–based analytical workflow.

Against this background, we conducted a retrospective analysis of patients with RVO-ME who received the standard three-injection anti-VEGF therapy regimen. Multimodal data were systematically collected prior to each injection, encompassing clinical information, imaging parameters (e.g., CMT), peripheral blood cell counts, and derived systemic inflammatory indices. Anatomical response at 6 months was utilized as the criterion for classifying patients into responders or non-responders. Following feature selection, nine widely adopted ML models were constructed and rigorously compared, and Bayesian optimization was employed for hyperparameter tuning to identify the model with optimal performance. Subsequently, SHAP analysis was then applied to provide multilevel interpretability, including global feature importance, variable-level interactions, and individualized prediction pathways. To further elucidate the mechanistic interplay between key predictors, we utilized Generalized Additive Models (GAMs) to explicitly map their complex, nonlinear interactions and to quantify their joint effects on treatment outcomes. Finally, to facilitate clinical translation, an interactive Shiny web tool was developed and deployed based on the optimal model, providing real-time visualization and decision support for anatomical outcome prediction (Figure 1). To the best of our knowledge, this represents the first study to combine peripheral blood-based systemic inflammatory indices and CMT for predicting the anatomical response to anti-VEGF therapy in RVO-ME, using interpretable ML approaches that unite predictive performance with mechanistic insights into the systemic inflammatory–retinal structural interplay.

## 2 Methods

### 2.1 Ethical consideration

This study was conducted in accordance with the principles of the Declaration of Helsinki and was approved by the Ethics Committee of the Affiliated Eye Hospital of Nanjing Medical University (approval number: 2024004). Due to the retrospective and non-interventional nature of the study, the requirement for informed consent was waived by the ethics committee.

### 2.2 Study design

This single-center, retrospective cohort study enrolled patients with RVO-ME who underwent a standardized loading regimen of three consecutive anti-VEGF intravitreal injections at the Affiliated Eye Hospital of Nanjing Medical University, between March 2021 and December 2024. The inclusion criteria were as follows: (1) age ≥18 years; (2) newly diagnosed with CRVO or BRVO accompanied by ME; (3) receipt of three consecutive injections of the same anti-VEGF agent (ranibizumab, conbercept, or aflibercept); and (4) availability of complete clinical information, CMT measurements, and concurrent peripheral blood cell count data during the treatment period. The exclusion criteria were as follows: (1) use of corticosteroids (e.g., dexamethasone implant [Ozurdex]), other anti-VEGF agents, or laser photocoagulation prior to or during treatment; (2) presence of other retinal diseases (e.g., DR, AMD, or glaucoma); (3) history of intraocular surgery or ocular trauma (4) significant media opacities (e.g., advanced cataract); that compromised OCTA image quality; (5) history of severe systemic, immune-mediated, or malignant diseases; (6) pregnancy or lactation; and (7) lack of OCTA imaging within 6 months following the third injection, precluding evaluation of ME response.

### 2.3 Data collection and outcome assessment

For each patient, multimodal data were collected prior to each of the three anti-VEGF injections and encompassed three core components: (1) clinical information, including age, sex, diabetes status, laterality of the affected eye, RVO type (CRVO or BRVO), type of anti-VEGF agent administered (ranibizumab, aflibercept, or conbercept), and best-corrected visual acuity (BCVA); (2) imaging parameters, with standardized anatomical images acquired using OCTA, from which CMT was extracted as a quantitative indicator; and (3) peripheral blood cell counts, with venous blood samples collected concurrently during the treatment period to determine neutrophil (N), lymphocyte (L), monocyte (M), and platelet (P) counts, from which six systemic inflammatory indices were calculated: NLR, PLR, LMR, SIRI, AISI, and SII.

All patients underwent OCTA follow-up within 6 months following the third anti-VEGF injection. Based on the anatomical response of ME, patients were categorized as responders or non-responders (Mori et al., 2020; Noma et al., 2020; Park et al., 2021). A responder was defined as having a CMT of <300 μm after the three consecutive injections, or a reduction of ≥10% compared to baseline, with no residual focal edema evident on imaging. Non-responders were further classified into two subtypes: (1) the recurrent subtype, defined as an initial resolution of ME after the three injections but subsequent recurrence during follow-up, evidenced by a CMT ≥300 μm or newly developed focal edema despite a CMT <300 μm; and (2) the refractory subtype, defined as a CMT reduction of <10% or even an increase after the three injections, or partial improvement that did not meet the response criteria. All imaging data and treatment outcomes were independently assessed in a masked fashion by two experienced retina specialists. Discrepancies were resolved by consensus through joint review.

### 2.4 Imaging and hematologic data acquisition and derivation of indices

Prior to each of the three anti-VEGF injections, all patients underwent standardized OCTA imaging and peripheral blood sampling. OCTA scans were acquired using the Optovue RTVue XR Avanti system (Optovue Inc., Fremont, CA, USA), obtaining 3 × 3 mm volumetric scans centered on the fovea before treatment. All scans were performed under uniform ambient lighting conditions following pharmacologic pupillary dilation. Images were included only if the quality index (Q-value) exceeded 6 and if free from significant motion artifacts or segmentation errors. CMT was defined as the maximum vertical distance from the retinal pigment epithelium to the internal limiting membrane in the central macular area. CMT values were initially obtained using the system’s automated segmentation algorithm. All measurements were then independently and blindly reviewed by two experienced retina specialists to verify segmentation accuracy. Manual correction was required infrequently, and all adjustments followed a standardized procedure. Any discrepancies between the two evaluators were resolved through consensus to ensure consistency and reliability of the final CMT values.

Peripheral venous blood samples were collected from the antecubital vein on the day of each injection using standard EDTA-K2 anticoagulant tubes and analyzed within 2 hours using a Sysmex XS-900i automated hematology analyzer (Sysmex Corporation, Kobe, Japan). Absolute counts of N, L, M, and P were recorded. All laboratory procedures were conducted by certified clinical laboratory technicians, and the hematology analyzer underwent routine daily calibration and quality control. Six systemic inflammatory indices were computed based on the absolute blood cell counts using the following standard formulas:

$$NLR=\frac{N}{L}$$

$$PLR=\frac{P}{L}$$

$$LMR=\frac{L}{M}$$

$$SIRI=\frac{N\times M\;}{L}$$

$$AISI=\frac{N\times M\times P\;}{L}$$

$$SII=\frac{P\times N\;}{L}$$

### 2.5 Intravitreal anti-VEGF injections

Prior to treatment, each patient selected an anti-VEGF agent based on clinical evaluation and financial considerations. The treating ophthalmologist provided a comprehensive explanation of the treatment objectives, potential risks, and alternative options, and written informed consent was obtained from each patient. Patients were instructed to instill 0.5% levofloxacin hydrochloride eye drops in the operative eye four times daily for 3 days preceding the injection. Topical anesthesia was administered using 0.4% oxybuprocaine hydrochloride eye drops. After meticulous aseptic preparation of the ocular surface, a lid speculum was used to maintain eyelid retraction. The conjunctival sac was disinfected with 5% povidone-iodine solution, followed by three irrigations with sterile saline. The injection site was performed at the pars plana, 3.5–4.0 mm posterior to the limbus. Intravitreal injection was performed perpendicularly into the vitreous cavity using a 1 mL syringe fitted with a sterile 30-gauge needle. After the injection, the needle was immediately withdrawn, and the injection site was gently compressed to prevent vitreous reflux or hemorrhage and to exclude acute complications. Postoperatively, tobramycin-dexamethasone ophthalmic ointment was applied to the conjunctival sac, and the eye was patched. Patients continued to instill 0.5% levofloxacin hydrochloride eye drops four times daily for 1 week to prevent postoperative infection.

### 2.6 Feature engineering and selection

All data processing and statistical analyses were performed using R software version 4.2.2 (R Foundation for Statistical Computing, Vienna, Austria). The outcome variable was defined as a binary factor, with “0” representing treatment responders and “1” representing non-responders. Categorical variables, including sex, diabetes status, eye laterality, RVO subtype, and anti-VEGF agent, were converted into factor variables. During model fitting, these factors were encoded as dummy indicator variables, with one category serving as the reference, so that no artificial ordinal relationships were introduced. For all numeric variables, suffixes “-0,” “-1,” and “-2” denoted measurements obtained prior to the first, second, and third anti-VEGF injections, respectively. To capture temporal trends, the min, max, and mean values of each numeric variable across these three time points were computed. Initial statistical analyses were conducted using the twogrps() function from the CBCgrps package (Zhang et al., 2017) to compare variable differences between responder and non-responder groups. Continuous variables were presented as mean ± standard deviation (SD) or median with interquartile range (IQR), depending on their distribution, and were compared between groups using the independent-samples t-test or the Mann-Whitney U test, as appropriate. Categorical variables were expressed as counts and percentages (n, %) and compared using the χ^2^ test or Fisher’s exact test. All tests were two-sided, and *P* values <0.05 were considered statistically significant. Before model development, the dataset was randomly partitioned into training and test sets in an 80:20 ratio. To mitigate multicollinearity, Spearman correlation matrices for numeric variables were calculated within the training set to identify pairs of highly correlated variables (|r| > 0.90 and *P* < 0.05). Stepwise regression based on Akaike information criterion (AIC) and variance inflation factor (VIF) analyses were subsequently used to further assess multicollinearity, and variables with VIF >10 were excluded. The remaining variables, together with those not forming highly correlated pairs, comprised the candidate feature set. The minimum and maximum values for each numeric variable were determined in the training set and used to perform min–max normalization separately for the corresponding variables in both the training and test sets. Finally, feature selection was performed on the normalized training set using a combination of least absolute shrinkage and selection operator (LASSO) regression and the Boruta algorithm to identify the core features for model development.

### 2.7 Model development and evaluation

Nine widely used ML models were developed using the training set, including gradient boosting tree models—extreme gradient boosting (XGBoost), light gradient boosting machine (LightGBM), and categorical boosting (CatBoost); ensemble learning models—adaptive boosting (AdaBoost) and random forest (RF); a linear model—elastic net logistic regression (ENLR); a kernel-based model—support vector machine (SVM); a distance-based model—k-nearest neighbors (KNN); and a neural network model—multilayer perceptron (MLP) (Badillo et al., 2020; Sarker, 2021). To improve model generalizability and robustness, Bayesian optimization was performed for hyperparameter tuning of each model within the training set (Snoek et al., 2012; Bates et al., 2024), with the objective of maximizing the area under the receiver operating characteristic curve (AUC) of the validation set during five-fold cross-validation. After identifying the optimal hyperparameter combination, each model was retrained on the entire training set, and the optimal classification threshold was determined based on the Youden index (Ruopp et al., 2008) calculated from the predicted probabilities. Model generalization performance was subsequently evaluated on the independent test set, which remained completely unseen during feature engineering, feature selection, hyperparameter tuning and threshold determinationto ensure that no data leakage occurred. Multiple metrics were employed to assess model performance, including accuracy, sensitivity, specificity, precision, and the F1 score (Sokolova and Lapalme, 2009). To comprehensively assess the reliability and clinical applicability of the predicted probabilities, receiver operating characteristic (ROC) curves were plotted, and corresponding AUC values were computed to evaluate discriminatory ability. Additionally, calibration curves and Brier scores were used to assess calibration, and decision curve analysis (DCA) was conducted to quantify the net clinical benefit of each model across a range of risk thresholds (Steyerberg et al., 2010; Vickers et al., 2019).

### 2.8 Model interpretability analysis

To enhance the interpretability of the models, SHAP analysis was performed on the ML model that demonstrated the highest performance on the test set. Interpretability analyses were conducted in R using the shapviz and fastshap packages. Global interpretability was assessed using feature importance rankings and beeswarm plots to visualize the overall contribution of each feature and to identify key predictors driving model decisions. Variable dependence plots were generated to illustrate the relationships between individual feature values and their corresponding SHAP values, incorporating color gradients to reflect feature interactions and overlaying GAM smoothers to uncover potential nonlinear effects. For local interpretability, force plots were constructed to visualize the prediction pathways for representative individuals, demonstrating how multivariate interactions contribute to individualized risk assessment and thereby enhancing the model’s clinical interpretability and potential applicability.

### 2.9 GAM modeling and interaction mechanism analysis

To further explore the combined effects of key features on anatomical response to anti-VEGF therapy in RVO-ME and assess potential intervention effects, we implemented a supplementary multi-level mechanistic analysis using GAMs. CMT-2, SII-min, and NLR-min were truncated at the 1st–99th percentiles to minimize the influence of outliers. Two GAMs were developed: a main-effects model including the three variables and relevant clinical covariates, and an extended model incorporating their pairwise interactions (CMT-2 × SII-min, CMT-2 × NLR-min, SII-min × NLR-min) to capture nonlinear relationships. The significance of the interaction structure was evaluated using a likelihood ratio test comparing the main-effects model with the full model, which yields a single overall *P*-value. Predictive probabilities from both models were used to generate ROC curves, with AUCs and 95% confidence intervals (CI) calculated and compared using the DeLong test. Optimal cut-off values for CMT-2, SII-min, and NLR-min were determined using the Youden index. Patients were subsequently stratified into different risk groups based on these thresholds, and the association between stratification and treatment outcome was assessed using Fisher’s exact test. To characterize the joint effects of variable combinations on nonresponse risk, two-dimensional grids were constructed for each variable pair, with all other covariates fixed at their median or reference levels. The GAM-predicted probabilities were used to generate contour risk maps, overlaid with threshold lines and patient distributions. Model uncertainty across the variable space was assessed using the delta method to derive standard errors (SE) on the probability scale, which were visualized as uncertainty contour plots. Finally, counterfactual simulations were performed in nonresponders by reducing SII-min or NLR-min from the 75th to the 25th percentile, and predicting the change in nonresponse probability using the GAM. Scenarios involving joint modulation of both markers were also evaluated, with median gains calculated in patients with positive probability gains, enabling quantification of individual benefit and identification of high-risk patients most likely to benefit from inflammatory modulation.

### 2.10 Shiny web-based interactive risk prediction platform

To facilitate individualized risk assessment and support clinical decision-making, an interactive web-based tool was constructed using the Shiny framework in R, based on the top-performing ML model identified during model development and evaluation. Within the graphical user interface, clinicians can input patient-specific feature values, and the system automatically returns the patient’s non-response prediction probability, model classification result, and the decision threshold currently applied. In addition, the platform generates feature importance ranking plots and individualized SHAP force plots, offering intuitive predictive insights and a transparent decision pathway, thereby enhancing the interpretability and practical utility of the model in clinical settings. To ensure consistency and reliability, the deployed model corresponds to the final version retrained on the full training set with optimized hyperparameters. All input data are processed using the same preprocessing pipeline applied during model training, thereby maintaining strict alignment between development and deployment stages.

## 3 Results

### 3.1 Patient characteristics and dataset partitioning

A total of 221 patients with RVO-ME (221 eyes) were initially screened, of whom 202 patients (202 eyes) met the predefined inclusion and exclusion criteria and were included in the final analysis. Based on anatomical response status at follow-up after three anti-VEGF injections, 106 patients (52.48%) were classified as responders, and 96 (47.52%) as non-responders. Supplementary Table S1 summarizes the baseline clinical characteristics, imaging parameters (CMT), peripheral blood cell counts, and systemic inflammatory indices for the two groups. There were no statistically significant differences in age, sex, diabetes status, laterality of the affected eye, RVO type, type of anti-VEGF agent administered, or BCVA between groups (*P* > 0.05). However, with respect to imaging parameters, the responder group exhibited significantly lower CMT measurements prior to the second and third injections, as well as lower minimum (min), maximum (max), and mean values during the treatment course compared to the non-responder group (*P* < 0.05). In addition, several systemic inflammatory indices showed significant group differences. Specifically, the responder group exhibited lower NLR, AISI, and SII values at various time points (prior to the first or third injection) and across statistical measures (min, max, and mean values) (*P* < 0.05).

To facilitate subsequent ML model development, all samples were randomly divided into a training set (n = 162) and a test set (n = 40) using an 80:20 split. The distribution of all input variables was systematically evaluated between the two sets to ensure baseline balance (Supplementary Table S2). No statistically significant differences were observed (*P* > 0.05), confirming that the random allocation process did not introduce selection bias and that the training and test sets were appropriately matched for model development.

### 3.2 Collinearity control and key feature selection

**FIGURE 2:**
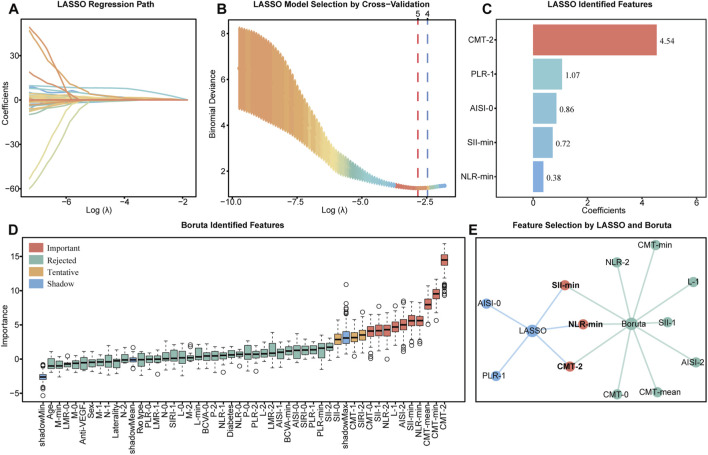
Feature selection by LASSO and Boruta. **(A–C)** LASSO coefficient profiles, cross-validation for λ selection, and non-zero features ranked by coefficients. **(D)** Boruta feature importance with selected variables (red). **(E)** Network plot showing overlap between LASSO (blue) and Boruta (green), with shared features in red.

Spearman correlation analysis was performed on all numerical variables within the training set (Supplementary Figure S1), identifying 24 feature pairs with high collinearity (|ρ| > 0.90, *P* < 0.05), indicating substantial multicollinearity (Supplementary Table S3). These correlated pairs were extracted to form an analysis subset, which was subjected to stepwise regression using response status as the dependent variable. This process yielded 17 representative features under collinearity constraint (Supplementary Table S4). Subsequently, VIF analysis was performed to further quantify multicollinearity, and variables with VIF >10 were excluded (Supplementary Figure S2). Ultimately, four features remained after collinearity control and were combined with non-redundant variables from the original dataset to form the candidate feature set. Feature selection was then independently performed on the candidate set using both LASSO regression and the Boruta algorithm. For LASSO, five-fold cross-validation was employed to identify the optimal regularization parameter λ, achieving the lowest mean squared error at λ = 0.058 (lambda.min), resulting in the retention of five features with non-zero coefficients (Figures 2A–C). The Boruta algorithm identified 10 stable and important features by comparing each with its respective shadow feature distribution (Figure 2D). By integrating the outputs of both methods, three overlapping features—CMT-2, NLR-min, and SII-min—were selected as key predictors. (Figure 2E). To further contextualize the relevance of these features, we evaluated their clinical distributions and found consistently higher median values in non-responders compared with responders (CMT-2: 305.50 vs. 250.00, *P* < 0.001; NLR-min: 1.89 vs. 1.62, *P* = 0.024; SII-min: 344.99 vs. 267.90, *P* = 0.014), supporting their role as important predictors of treatment response.

### 3.3 Model comparison and generalization performance evaluation

Using the three core features identified during feature selection, nine widely adopted ML models were constructed on the training set: XGBoost, LightGBM, CatBoost, AdaBoost, RF, ENLR, SVM, KNN, and MLP. Hyperparameter tuning for all models was performed via five-fold cross-validation with Bayesian optimization. The optimal parameter sets are summarized in Supplementary Table S5. Final performance evaluations were conducted on models retrained using the entire training set, with the optimal classification threshold determined within the training set applied (Table 1).

**TABLE 1:** Optimal classification thresholds from training set.

Model	XGBoost	LightGBM	CatBoost	AdaBoost	RF	ENLR	SVM	KNN	MLP
Threshold	0.499	0.387	0.489	0.474	0.431	0.553	0.580	0.500	0.341

**FIGURE 3:**
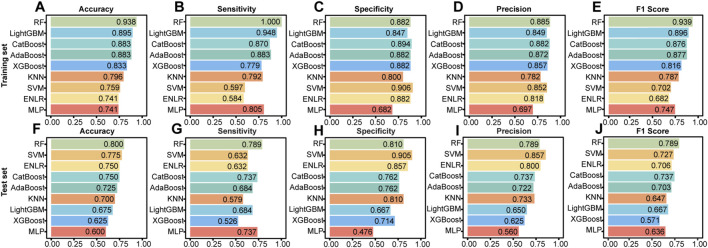
Performance of nine ML models evaluated by five metrics on training **(A–E)** and test **(F–J)** sets. Bars indicate scores from final retrained models with optimized hyperparameters.

**FIGURE 4:**
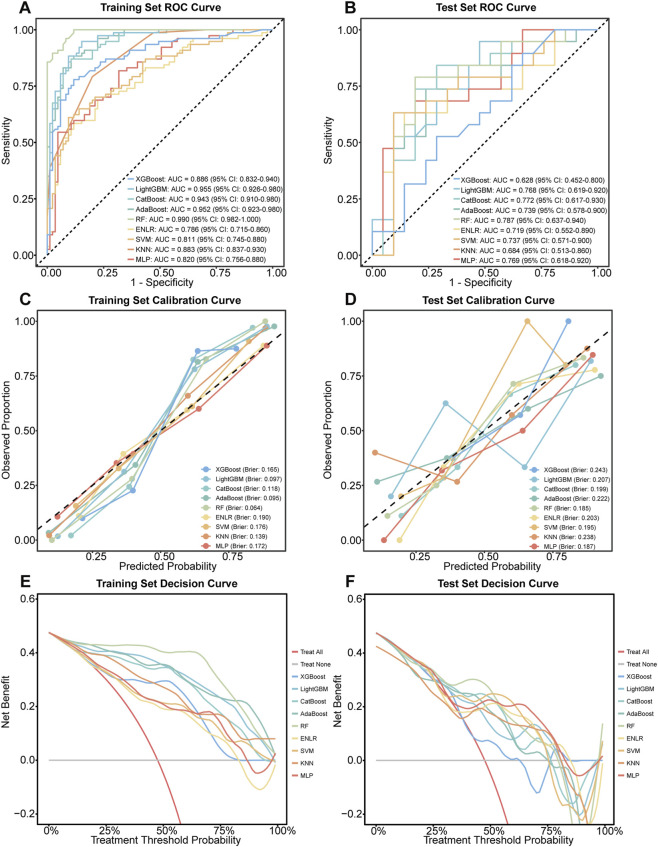
Comparative discrimination, calibration, and decision-analytic performance. **(A,B)** ROC curves with AUC and 95% confidence intervals. **(C,D)** Calibration curves with Brier scores. **(E,F)** Decision curve analysis of clinical net benefit across threshold probabilities.

Based on comprehensive evaluation metrics, the RF model demonstrated superior overall performance across both the training and independent test sets (Figures 3, 4). In the test set, the RF model achieved the highest accuracy (0.800), outperforming the next best models, SVM (accuracy = 0.775) and ENLR (accuracy = 0.750). Regarding classification balance, the RF model exhibited well-balanced sensitivity (0.789) and specificity (0.810) in the test set, surpassing all other models on these two critical indicators. Additionally, it yielded a high precision (0.789), reflecting strong positive predictive performance. The F1 score of 0.789 further highlighted the model’s ability to effectively balance precision and recall (Figures 3F–J). With respect to prediction reliability, the RF model attained the highest AUC (0.787), indicating outstanding discriminatory capacity. It also had the lowest Brier score (0.185), with a calibration curve closely aligned with the ideal fit line, indicating good agreement between predicted probabilities and actual observations. DCA further supported the clinical applicability of the RF model, showing consistent net benefit across a treatment threshold range of 0.2–0.5, surpassing the other models (Figures 4B,D,F). Confusion matrices for all nine ML models in both the training and test sets are provided in Supplementary Figure S3, offering a visual summary of classification outcomes.

### 3.4 Contributions of key features and individualized prediction pathways

**FIGURE 5:**
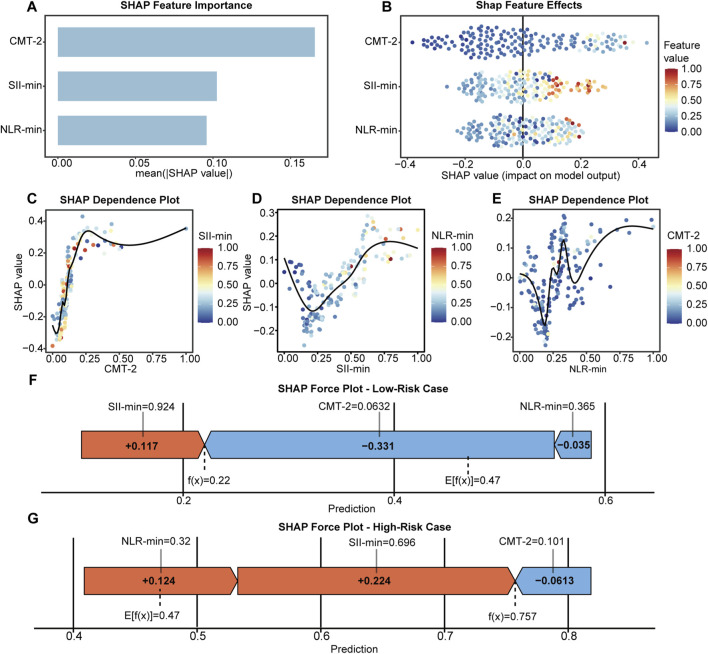
SHAP-based interpretation of the RF model. **(A,B)** Global feature importance and summary plot. **(C–E)** Dependence plots for CMT-2, SII-min, and NLR-min. **(F,G)** Force plots for representative responder and non-responder, illustrating individual-level feature contributions.

SHAP-based interpretability analysis was performed on the RF model that demonstrated superior performance in the test set. The feature importance bar plot revealed that among the three selected predictors, CMT-2 exhibited the highest mean SHAP value, contributing more overall than SII-min and NLR-min (Figure 5A). The SHAP beeswarm plot further illustrated the directional impact of each feature across its value range. The SHAP values for CMT-2 were predominantly negative, suggesting that lower CMT-2 values generally drove the model’s predictions toward the responder class in most samples. In contrast, higher SII-min and NLR-min values were generally associated with positive SHAP values, indicating that elevated systemic inflammation tended to drive predictions toward non-responder class (Figure 5B).

The SHAP variable dependence plots uncovered nonlinear contribution patterns and interaction effects among the three selected features. For CMT-2, an “L-shaped” negative relationship was observed, in which low values exerted a strong negative influence on model predictions, while the marginal impact diminished as values increased (Figure 5C). SII-min exhibited an approximately linear positive association with its SHAP values, indicating that higher levels consistently drove predictions toward the non-responder class (Figure 5D). The relationship between NLR-min and SHAP values was nonlinear, with marked fluctuations in the mid-range (approximately 0.2–0.4) and a more stable pattern at both lower and higher extremes (Figure 5E). Moreover, the color gradient in these plots reflected interaction effects between variables. Specifically, in samples with higher SII-min, the magnitude of the positive SHAP values for CMT-2 became more pronounced. Similarly, higher NLR-min values further increased the SHAP values of SII-min. Additionally, elevated CMT-2 values were associated with more positive SHAP values for NLR-min (Figures 5C–E).

At the local interpretability level, SHAP force plots were utilized to visualize individualized prediction pathways for two representative cases. In the case depicted in Figure 5F, a low CMT-2 value (0.0632) served as the primary negative driver, substantially reducing the prediction score (−0.331). In contrast, a high SII-min value (0.924) contributed positively (+0.117), with the overall prediction favoring the responder class. Conversely, the case shown in Figure 5G exhibited elevated NLR-min and SII-min values (0.32 and 0.696, respectively), which together contributed a total positive SHAP value of approximately +0.348, ultimately shifting the prediction toward the non-responder class. These results highlight the robust local interpretability of the model and its ability to uncover individualized predictive mechanisms driven by multivariable interactions.

### 3.5 Interaction effects and counterfactual intervention analysis

**FIGURE 6:**
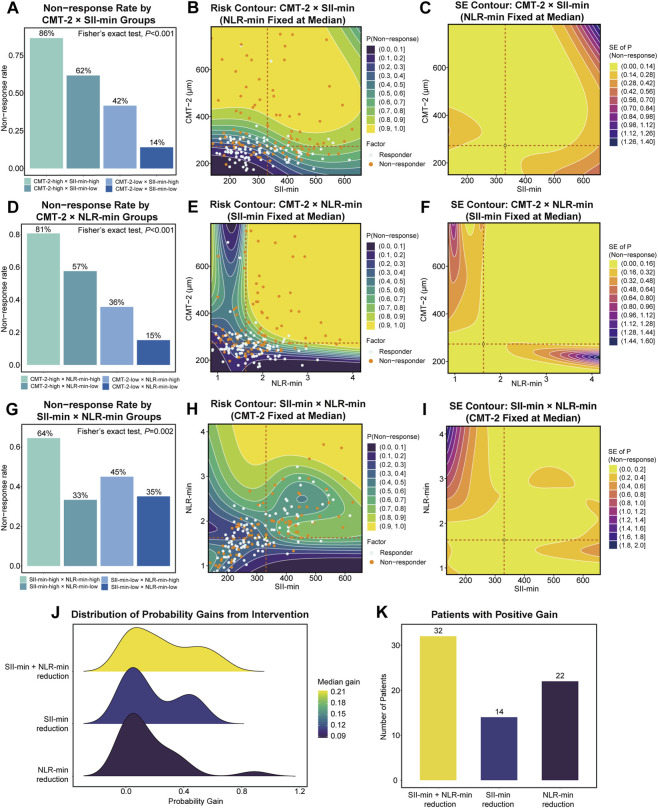
Interaction effects and counterfactual intervention analysis. **(A,D,G)** Non-response rates by stratified subgroups (Fisher’s exact test). **(B,E,H)** Two-dimensional contour risk maps for pairwise interactions. **(C,F,I)** Corresponding uncertainty contour maps. **(J)** Ridgeline plots of probability gains from counterfactual simulations. **(K)** Number of non-responders predicted to benefit under each intervention scenario.

The interaction structure significantly improved overall model fit (*P* = 0.006). In line with this improvement, ROC curve analysis showed that the interaction model outperformed the main-effects model, with an AUC of 0.863 versus 0.825 (*P* = 0.016) (Supplementary Figure S4). Based on the interaction model, the optimal clinical stratification thresholds for CMT-2, SII-min, and NLR-min were determined using the Youden index, yielding values of 273.00, 330.75, and 1.63, respectively. Stratification revealed clear risk gradients: when both CMT-2 and SII-min were high, the non-response rate reached 86%, compared with 14% when both were low; with one high and one low, the non-response rates were intermediate (62% and 42%), with significant differences across groups (*P* < 0.001) (Figure 6A). Similar interaction effects were observed for CMT-2 × NLR-min and SII-min × NLR-min (Figures 6D,G). Contour risk maps further illustrated the distribution of non-response probabilities across pairwise variable combinations. In the CMT-2 and SII-min map, risk smoothly transitioned from a deep-blue low-risk zone in the lower left corner (both low) to a yellow high-risk zone in the upper right corner (both high) (Figure 6B). The actual distribution of responders (blue dots) and non-responders (yellow dots) was highly consistent with the model’s predicted risk landscape. Similar patterns were observed for the other two variable combinations, where simultaneous elevation of both variables was associated with a sharp increase in non-response risk (Figures 6E,H). SE contour plots indicated stable estimates in data-dense regions and greater uncertainty at the boundaries (Figures 6C,F,I). Counterfactual simulations showed median probability gains of 0.110 and 0.088 when SII-min or NLR-min were reduced from the 75th to 25th percentile, respectively, while joint intervention produced the largest benefit (median gain = 0.215) (Figure 6J), improving outcomes in 32 non-responders compared with 14 and 22 patients for single-marker interventions (Figure 6K). Detailed results are presented in Supplementary Table S6.

### 3.6 Clinical deployment of the shiny web-based prediction platform

**FIGURE 7:**
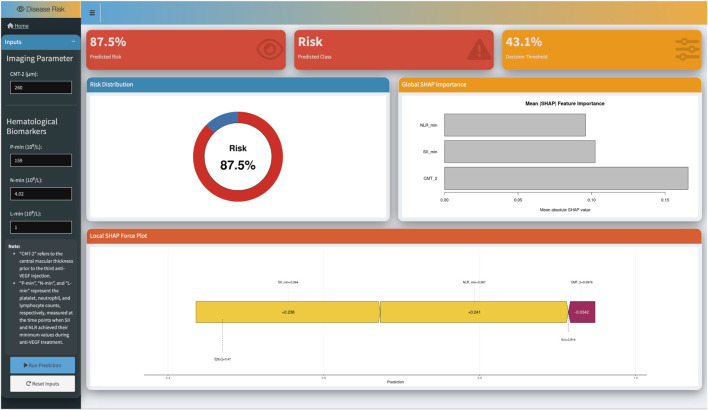
Screenshot of the web-based interactive tool for individualized prediction of treatment response. Users input CMT and hematologic parameters to obtain individualized predictions, SHAP explanations, and feature contributions.

To enhance the clinical applicability of the model and facilitate interactive visualization, a web-based risk prediction platform was developed and deployed using the Shiny framework, based on the RF model that demonstrated superior performance in the test set (Figure 7). This platform can be accessed via the following link: https://huginnlee.shinyapps.io/RVO_ME_Predict/. The user interface features a manual input module for key variables, including central macular thickness prior to the third injection (CMT-2) and peripheral blood cell counts: minimum platelet (P-min), neutrophil (N-min), and lymphocyte (L-min) values. The system automatically calculates derived systemic inflammatory indices, namely NLR-min and SII-min, and integrates them into the model to generate individualized prediction results. The platform provides real-time output consisting of the following components: (1) Predicted response probability and corresponding classification label; (2) The current decision threshold applied by the model; (3) A global SHAP feature importance ranking plot; (4) A local SHAP force plot visualizing the positive and negative contributions of each feature to the final prediction.

## 4 Discussion

RVO is a prevalent vision-threatening retinal vascular disease caused by impaired venous blood flow and increased intravascular pressure. The resultant hemodynamic disturbances lead to localized retinal ischemia and hypoxia, which in turn trigger excessive expression of VEGF. This cascade compromises the integrity of the BRB, leading to fluid leakage and the development of ME. However, the progression and clinical manifestations of RVO are not exclusively governed by hemodynamic mechanisms. Accumulating evidence highlights the critical role of systemic inflammation in the pathophysiology of RVO. Elevated systemic inflammatory status can upregulate circulating pro-inflammatory cytokines, promote platelet and leukocyte adhesion, exacerbate local endothelial dysfunction, and increase vascular permeability, thereby establishing a persistent inflammatory microenvironment (Kazantzis et al., 2022; Noma et al., 2022). This interplay between systemic and local inflammatory processes not only perpetuates ME but may also account for the observed heterogeneity in anatomical responses to anti-VEGF therapy. Building on this foundation, the present study systematically integrated the CMT, a direct indicator of ME severity, with peripheral blood-based systemic inflammatory indices to develop and validate an ML framework for predicting anatomical response of RVO-ME patients following a standardized three-injection anti-VEGF therapy regimen. Through a multi-stage feature selection process, CMT-2, NLR-min, and SII-min were identified as the most predictive features for the model. The RF model incorporating these three core features demonstrated superior predictive performance and clinical generalizability compared to eight other widely adopted ML models. SHAP and GAMs analysis further provided mechanistic insights by quantifying the contribution and interaction of each variable. The Shiny-based interactive risk prediction platform, implemented using the optimal model, balances performance and practicality, providing a convenient, transparent, and user-friendly decision-support tool to facilitate individualized clinical decision-making.

A key finding of this study is that the CMT measured prior to the third anti-VEGF injection (CMT-2) emerged as the most discriminative feature for predicting anatomical response. As a direct anatomical parameter quantifying ME, the clinical utility of CMT in evaluating anti-VEGF treatment efficacy has been extensively documented in previous studies. For instance, Chang et al. reported that a higher baseline CMT significantly increased the risk of ME recurrence in RVO patients (Chang et al., 2025). Similarly, Zhou et al. found demonstrated that a reduction in CMT of exceeding 37% 2 weeks after the first anti-VEGF injection was a robust predictor of complete resolution of RVO-ME within 6 months (Zhou et al., 2022). In our study, we further substantiated that CMT-2, compared with baseline or early change indicators, exhibited superior predictive value. A lower CMT-2 may reflect a rapid and substantial anatomical recovery during the early stages of treatment, potentially characterizing a phenotype highly responsive to anti-VEGF therapy. SHAP analysis further corroborated this finding, revealing a nonlinear, “L-shaped” pattern in which CMT-2 exerted a dominant negative contribution to model outputs. Specifically, lower CMT-2 values were strongly associated with an increased probability of treatment response, while this predictive effect plateaued at higher CMT-2 values. These findings indicate that early, substantial anatomical improvement may serve as a critical biomarker for favorable therapeutic prognosis in RVO-ME.

Beyond the imaging marker CMT-2, this study identified NLR-min and SII-min during the course of anti-VEGF treatments as important predictors of anatomical response. NLR reflects the balance between neutrophil-mediated innate immune activation and lymphocyte-mediated adaptive immune regulation, while SII integrates neutrophil, lymphocyte, and platelet counts to provide a more comprehensive assessment of systemic inflammation and platelet activation (Chen et al., 2017; Buonacera et al., 2022). Previous studies have established a strong association between elevated levels of these indices and both the occurrence and severity of RVO. For example, Zuo et al. demonstrated that both NLR and SII were significantly elevated in RVO patients compared to healthy controls, with SII exhibiting superior predictive power for RVO risk (Zuo et al., 2023). Doğan et al. further reported that RVO patients with serous retinal detachment had significantly higher NLR and SII levels than those without (Doğan et al., 2023). Our SHAP analysis revealed that higher NLR-min and SII-min values were strongly associated with an increased risk of anatomical non-response. These findings suggest that a persistent or insufficiently suppressed systemic inflammatory state may serve as a robust surrogate for the intraocular proinflammatory milieu. Such systemic inflammation could exacerbate local inflammation processes in the macular region by upregulating pro-inflammatory cytokines such as IL-6 and TNF-α, leading to BRB breakdown and enhanced vascular leakage, ultimately reducing the therapeutic efficacy of anti-VEGF agents. Importantly, this study utilized the minimum values of these systemic inflammatory indices recorded throughout treatment, in contrast to the single baseline measurements commonly used in most previous studies. These minima may more accurately reflect the optimal degree of inflammation suppression achieved with continuous therapeutic intervention. Elevated minimum values, therefore, likely represent a more recalcitrant and less reversible systemic inflammatory tendency, serving as a crucial driver of persistent ME and reducing the likelihood of a favorable anatomical response to anti-VEGF therapy.

More importantly, this study revealed that CMT-2, NLR-min, and SII-min do not function as independent predictors of anatomical response; instead, they exhibit significant interactive effects. SHAP dependence analysis demonstrated nonlinear biases and interaction patterns, which were corroborated by a GAM with explicit pairwise interaction terms. The interaction-based GAM achieved significantly higher discriminative performance than the main-effect-only model, underscoring that structural burden and systemic inflammation are not merely additive but act synergistically to amplify the risk of non-response. Further analyses using Youden index-derived thresholds and contour risk maps provided intuitive evidence for this mechanism: a concordant-high configuration was associated with a markedly increased probability of non-response, whereas a concordant-low configuration corresponded to substantially reduced risk, with discordant patterns yielding intermediate risk. This risk gradient mirrored the color transitions and marginal effects observed in the SHAP dependence plots. Under heightened systemic inflammation, reductions in CMT-2 conferred less benefit, suggesting that heightened systemic inflammation may attenuate the beneficial impact of local anatomical improvements on anatomical outcomes. Conversely, when both CMT-2 and systemic inflammation increased, structural burden and inflammatory activation jointly increased the predicted probability of non-response. Moreover, the concomitant elevation of SII-min and NLR-min further magnified the risk of unfavorable outcomes, indicating that a systemic inflammatory state characterized by both increased inflammatory activation and impaired immune regulation may represent a potential mechanism underlying resistance to anti-VEGF therapy. Under such conditions, enhanced pro-inflammatory cytokine release, reduced immunosuppressive activity, and increased platelet-mediated microvascular injury may collectively contribute to the persistence of macular anatomical edema, rendering it refractory to reversal.

From a modeling perspective, this study systematically compared nine widely adopted ML models and confirmed the superior performance of the RF model in integrating key systemic inflammatory indices and CMT for anatomical response prediction. As an ensemble learning method, RF constructs multiple decision trees and aggregates their outputs via majority voting, enabling it to effectively capture complex nonlinear interactions among variables. Furthermore, RF is particularly well-suited for modeling on small datasets and in the presence of noise, making it highly applicable to real-world clinical environments (Han et al., 2021). The potential utility of RF in retinal vascular disease prediction has been validated in previous studies. For instance, Zhang et al. employed an RF model to predict treatment response to anti-VEGF therapy in patients with neovascular AMD, achieving an AUC of 0.91 in the validation cohort, significantly outperforming other ML models (Zhang et al., 2025). These findings underscore the clinical relevance and translational feasibility of RF for individualized prognostic modeling in retinal vascular diseases.

The RF model developed in this study demonstrated considerable potential for clinical translation. Currently, the evaluation of therapeutic response to anti-VEGF therapy in patients with RVO-ME predominantly relies on post-treatment follow-up, with a notable lack of early, individualized prediction tools. Existing ML and deep-learning studies, whether based on structural and physiological parameters or high-dimensional imaging data, have not incorporated systemic inflammation into their predictive frameworks, nor have they offered interpretability methods to elucidate how systemic inflammatory activity interacts with macular structural changes (Zhang L. et al., 2024; Zhang Y. et al., 2024; Liang et al., 2025). In this study, we integrated readily accessible systemic inflammatory indices with CMT-2 for the first time, achieving a streamlined feature set with improved interpretability. Our model requires only pre-treatment CMT measurements and concurrent peripheral blood cell counts prior to three anti-VEGF injections, rendering data acquisition both straightforward and cost-effective, thereby supporting its feasibility for broad clinical implementation. The deployed Shiny-based web tool allows clinicians to conveniently input patient-specific parameters to rapidly obtain predicted response probabilities and accompanying interpretability insights. This not only facilitates the early identification of high-risk non-responders and enables timely optimization of treatment strategies, but also enhances physician-patient communication, improving patient understanding of treatment expectations and adherence. For example, in patients with low CMT-2 but elevated NLR-min or SII-min values, the model indicates that persistent systemic inflammation may offset the favorable effect of early anatomical improvement, suggesting the potential need for adjunctive anti-inflammatory or corticosteroid therapy. Conversely, patients with concurrently elevated CMT-2, NLR-min, and SII-min are identified as being at high risk due to the dual contribution of anatomical burden and systemic inflammation, warranting early implementation of intensified treatment strategies, such as combination with intravitreal steroid implants or shortened treatment and follow-up intervals, to achieve more effective disease control. Crucially, counterfactual simulation analyses provided quantitative support for the feasibility of these strategies. Simulated reductions in either SII-min or NLR-min alone produced measurable improvements in predicted response probabilities, whereas combined modulation produced synergistic gains and markedly expanded the number of patients likely to benefit. These findings underscore the clinical value of systemic inflammation as a modifiable therapeutic target and provide a strong mechanistic and methodological foundation for its integration into personalized management of RVO-ME.

This study has several limitations that warrant acknowledgment. First, the single-center retrospective design and limited overall sample size constrain the generalizability of the model. Although rigorous internal validation and cross-validation were performed to mitigate selection bias, the independent test set remains too small to support a fully robust evaluation of the ML model. Therefore, external validation in larger multicenter prospective cohorts is essential to further establish the stability and applicability of the model across diverse clinical settings. Second, anatomical response was defined based exclusively on changes in CMT, without incorporating improvements in visual function as a co-primary outcome. While the resolution of ME provides an anatomical basis for visual recovery, structural and functional improvements do not always occur concurrently. Future research should aim to develop multi-objective predictive models that integrate both anatomical and functional endpoints to more comprehensively reflect real-world treatment goals. Finally, the input features used in this study were primarily limited to CMT and peripheral blood cell counts, excluding higher-dimensional imaging features such as retinal blood flow density, hyperreflective foci, or cyst size. In addition, during the initial feature-selection process, several other predictors with potential biological relevance, apart from the three features jointly identified by LASSO and Boruta, may also contribute incremental value to the model. Future studies with larger sample sizes may consider evaluating broader feature sets and alternative feature-selection strategies, as well as integrating deep learning-based imaging representations to enrich the predictor space and further enhance model performance.

In summary, this study successfully developed and validated an interpretable ML framework that integrates peripheral blood-based systemic inflammatory indices with CMT to predict anatomical response to anti-VEGF therapy in patients with RVO-ME. Through SHAP analysis and interactive GAM-based approaches, we uncovered the synergistic interplay between systemic inflammation and retinal structural burden, thereby elucidating the potential mechanisms driving treatment heterogeneity. The RF model, implemented within an interactive clinical platform, may serve as a valuable tool to facilitate early risk stratification and personalized therapeutic decision-making. Moreover, counterfactual simulations highlighted the substantial role of systemic inflammation as a modifiable therapeutic target, effectively bridging predictive modeling with actionable clinical intervention strategies. Although further prospective validation remains warranted, this study convincingly demonstrates the unique value of interpretable ML in both mechanistic discovery and precision management, offering a broadly generalizable paradigm for the intelligent and individualized treatment of RVO-ME and other retinal vascular diseases.

## Data Availability

The raw data supporting the conclusions of this article will be made available by the authors, without undue reservation.

## Glossary

AdaBoost: Adaptive boosting
AIC: Akaike information criterion
AISI: Aggregate index of systemic inflammation
AMD: Age-related macular degeneration
AUC: Area under the receiver operating characteristic curve
BCVA: Best-corrected visual acuity
BRB: Blood-retinal barrier
BRVO: Branch retinal vein occlusion
CatBoost: Categorical boosting
CI: Confidence intervals
CMT: Central macular thickness
CRVO: Central retinal vein occlusion
DR: Diabetic retinopathy
DCA: Decision curve analysis
ENLR: Elastic net logistic regression
GAM: Generalized additive model
IL-6: Interleukin-6
IQR: Interquartile range
KNN: K-nearest neighbors
L: Lymphocyte
LASSO: Least absolute shrinkage and selection operator
LightGBM: Light gradient boosting machine
LMR: Lymphocyte-to-monocyte ratio
M: Monocyte
ME: Macular edema
ML: Machine learning
MLP: Multilayer perceptron
N: Neutrophil
NLR: Neutrophil-to-lymphocyte ratio
OCT: Optical coherence tomography
OCTA: OCT angiography
P: Platelet
PLR: Platelet-to-lymphocyte ratio
RF: Random forest
ROC: Receiver operating characteristic
RVO: Retinal vein occlusion
SD: Standard deviation
SE: standard errors
SHAP: SHapley Additive exPlanations
SII: Systemic immune-inflammation index
SIRI: Systemic inflammation response index
SVM: Support vector machine
TNF-α: Tumor necrosis factor-alpha
VEGF: Anti-vascular endothelial growth factor
VIF: Variance inflation factor
XGBoost: Extreme gradient boosting
