# Supplementary material for: Integration of peripheral blood-based systemic inflammatory indices and retinal imaging using interpretable machine learning for predicting anti-VEGF treatment response in macular edema secondary to retinal vein occlusion
Source: Front Cell Dev Biol. 2025 Dec 29;13:1732963. doi: 10.3389/fcell.2025.1732963 (PMC12794559; doi:10.3389/fcell.2025.1732963)
Supplement: Supplementary file 1 [file DataSheet1.pdf]

Supplementary Material

1 Supplementary Tables

Table S1 Comparison of characteristics between responders and non-responders with RVO-ME.

Variables	Total (n = 202)	Responder (n = 106)	Non-responder (n = 96)	*P*-value
Sex, n (%)				0.662
Male	99 (49.01)	54 (50.94)	45 (46.88)	
Female	103 (50.99)	52 (49.06)	51 (53.12)	
Laterality, n (%)				0.994
RE	102 (50.50)	53 (50.00)	49 (51.04)	
LE	100 (49.50)	53 (50.00)	47 (48.96)	
RVO-Type, n (%)				0.221
BRVO	146 (72.28)	81 (76.42)	65 (67.71)	
CRVO	56 (27.72)	25 (23.58)	31 (32.29)	
Anti-VEGF, n (%)				0.955
Ranibizumab	142 (70.30)	74 (69.81)	68 (70.83)	
Conbercept	48 (23.76)	26 (24.53)	22 (22.92)	
Aflibercept	12 (5.94)	6 (5.66)	6 (6.25)	
Diabetes, n (%)				0.233
No	183 (90.59)	99 (93.40)	84 (87.50)	
Yes	19 (9.41)	7 (6.60)	12 (12.50)	
Age	60.50 (52.25, 70.00)	60.50 (53.00, 69.00)	60.50 (52.00, 70.00)	0.987
BCVA-0	0.82 (0.52, 1.23)	0.92 (0.52, 1.30)	0.82 (0.52, 1.00)	0.341
BCVA-1	0.60 (0.40, 0.92)	0.60 (0.30, 1.00)	0.60 (0.40, 0.84)	0.940
BCVA-2	0.60 (0.30, 0.92)	0.52 (0.24, 0.92)	0.60 (0.30, 0.82)	0.619
BCVA-min	0.40 (0.22, 0.82)	0.46 (0.22, 0.90)	0.40 (0.30, 0.70)	0.930
BCVA-max	0.92 (0.60, 1.37)	0.96 (0.60, 1.40)	0.92 (0.60, 1.30)	0.585
BCVA-mean	0.64 (0.45, 1.06)	0.68 (0.41, 1.13)	0.64 (0.48, 0.94)	0.840
CMT-0, µm	483.00 (359.00, 633.75)	451.00 (346.00, 603.50)	515.50 (392.75, 646.50)	0.054
CMT-1, µm	284.00 (254.25, 359.00)	273.00 (236.25, 335.50)	292.00 (262.75, 375.50)	0.002^*
CMT-2, µm	268.00 (243.00, 317.00)	250.00 (225.25, 285.25)	305.50 (264.00, 427.75)	$< 0.001^*$
CMT-min, µm	258.50 (234.50, 289.00)	246.00 (216.25, 270.25)	273.50 (250.25, 302.75)	$< 0.001^*$
CMT-max, µm	508.50 (385.00, 672.50)	483.00 (360.25, 634.75)	574.50 (441.00, 702.50)	0.017^*
CMT-mean, µm	364.33 (308.50, 456.33)	331.50 (291.67, 412.42)	397.17 (333.25, 484.42)	$< 0.001^*$
N-0, 10^9/L	3.94 (3.18, 4.75)	3.70 (3.11, 4.48)	4.11 (3.28, 4.92)	0.064
N-1, 10^9/L	3.75 (2.98, 4.56)	3.67 (3.06, 4.46)	3.80 (2.93, 4.63)	0.539
N-2, 10^9/L	3.68 (3.09, 4.42)	3.74 (3.09, 4.21)	3.64 (3.13, 4.62)	0.421
N-min, 10^9/L	3.34 (2.68, 3.97)	3.20 (2.64, 3.96)	3.44 (2.80, 3.97)	0.198
N-max, 10^9/L	4.38 (3.66, 5.28)	4.30 (3.64, 5.05)	4.45 (3.68, 5.44)	0.333

N-mean, 10^9/L	3.86 (3.18, 4.54)	3.82 (3.18, 4.45)	3.97 (3.20, 4.68)	0.220
L-0, 10^9/L	1.86 ± 0.52	1.88 ± 0.52	1.85 ± 0.53	0.667
L-1, 10^9/L	1.90 ± 0.53	1.94 ± 0.59	1.85 ± 0.47	0.258
L-2, 10^9/L	1.86 (1.51, 2.30)	1.83 (1.53, 2.36)	1.86 (1.48, 2.19)	0.421
L-min, 10^9/L	1.64 ± 0.44	1.66 ± 0.46	1.62 ± 0.43	0.460
L-max, 10^9/L	2.14 ± 0.54	2.19 ± 0.57	2.09 ± 0.50	0.200
L-mean, 10^9/L	1.89 ± 0.47	1.92 ± 0.50	1.85 ± 0.43	0.272
M-0, 10^9/L	0.36 (0.28, 0.45)	0.35 (0.28, 0.42)	0.36 (0.29, 0.45)	0.216
M-1, 10^9/L	0.37 (0.30, 0.44)	0.37 (0.30, 0.45)	0.37 (0.31, 0.43)	0.942
M-2, 10^9/L	0.38 (0.30, 0.47)	0.36 (0.29, 0.47)	0.39 (0.30, 0.47)	0.539
M-min, 10^9/L	0.32 (0.26, 0.38)	0.31 (0.26, 0.36)	0.33 (0.26, 0.39)	0.493
M-max, 10^9/L	0.42 (0.35, 0.55)	0.41 (0.35, 0.54)	0.42 (0.36, 0.54)	0.559
M-mean, 10^9/L	0.37 (0.31, 0.47)	0.37 (0.30, 0.45)	0.38 (0.32, 0.47)	0.558
P-0, 10^9/L	188.00 (158.25, 220.00)	180.50 (152.25, 207.50)	198.00 (161.00, 227.25)	0.037*
P-1, 10^9/L	191.00 (152.25, 221.75)	190.00 (152.50, 212.75)	193.00 (152.00, 232.25)	0.296
P-2, 10^9/L	184.50 (154.25, 222.75)	179.00 (154.25, 221.50)	191.00 (156.25, 225.75)	0.308
P-min, 10^9/L	165.00 (135.00, 192.75)	163.00 (134.00, 187.50)	170.50 (141.75, 203.25)	0.191
P-max, 10^9/L	208.00 (171.00, 239.75)	203.00 (170.25, 230.00)	220.00 (177.50, 261.75)	0.060
P-mean, 10^9/L	188.50 (158.42, 216.58)	184.50 (155.00, 209.50)	196.00 (162.00, 225.75)	0.082
NLR-0	2.09 (1.67, 2.74)	2.03 (1.61, 2.43)	2.22 (1.83, 2.91)	0.063
NLR-1	2.04 (1.58, 2.61)	1.93 (1.56, 2.48)	2.11 (1.68, 2.74)	0.145
NLR-2	1.94 (1.61, 2.51)	1.86 (1.53, 2.38)	2.05 (1.69, 2.68)	0.046*
NLR-min	1.75 (1.43, 2.09)	1.62 (1.42, 1.98)	1.89 (1.47, 2.17)	0.024*
NLR-max	2.52 (1.93, 3.18)	2.40 (1.91, 3.11)	2.72 (2.05, 3.29)	0.146
NLR-mean	2.16 (1.66, 2.57)	2.00 (1.63, 2.51)	2.30 (1.76, 2.70)	0.042*
PLR-0	101.59 (82.00, 123.15)	98.26 (81.73, 117.52)	110.27 (82.38, 132.77)	0.146
PLR-1	100.69 (80.01, 122.32)	95.86 (82.06, 119.75)	105.04 (79.4, 128.88)	0.244
PLR-2	100.62 (83.40, 121.31)	100.00 (78.77, 118.24)	103.61 (90.61, 125.69)	0.106
PLR-min	86.42 (67.96, 101.69)	83.11 (67.43, 95.89)	92.80 (68.70, 109.18)	0.127
PLR-max	117.93 (100.00, 146.07)	115.55 (96.54, 138.17)	122.55 (103.77, 161.35)	0.039*
PLR-mean	102.52 (85.86, 122.52)	97.86 (83.04, 117.93)	105.83 (87.58, 128.57)	0.071
LMR-0	4.93 (3.97, 6.40)	5.07 (4.05, 6.37)	4.80 (3.85, 6.39)	0.310
LMR-1	4.96 (4.00, 6.15)	4.93 (4.08, 6.15)	5.08 (3.95, 6.02)	0.707
LMR-2	5.04 (3.92, 6.07)	5.26 (4.23, 6.24)	4.94 (3.86, 5.81)	0.139
LMR-min	4.26 (3.44, 5.13)	4.34 (3.44, 5.21)	4.18 (3.43, 5.12)	0.532
LMR-max	5.92 (4.73, 7.25)	5.96 (4.84, 7.16)	5.62 (4.61, 7.25)	0.540
LMR-mean	5.06 (4.14, 6.05)	5.12 (4.24, 6.09)	5.02 (4.05, 5.94)	0.347
SIRI-0	0.78 (0.54, 1.13)	0.75 (0.53, 1.02)	0.89 (0.55, 1.27)	0.061
SIRI-1	0.76 (0.53, 1.04)	0.71 (0.53, 1.02)	0.77 (0.52, 1.05)	0.438
SIRI-2	0.71 (0.54, 1.00)	0.67 (0.54, 0.86)	0.78 (0.54, 1.11)	0.090
SIRI-min	0.57 (0.44, 0.78)	0.55 (0.44, 0.73)	0.61 (0.44, 0.84)	0.191
SIRI-max	0.99 (0.71, 1.38)	0.93 (0.70, 1.27)	1.01 (0.72, 1.66)	0.131

SIRI-mean	0.79 (0.58, 1.06)	0.73 (0.58, 0.98)	0.84 (0.58, 1.17)	0.088
AISI-0	144.80 (98.81, 220.57)	133.45 (90.05, 187.72)	165.64 (103.10, 272.79)	0.008*
AISI-1	132.03 (94.03, 208.81)	127.16 (89.71, 183.83)	134.88 (97.14, 248.65)	0.145
AISI-2	127.25 (93.65, 202.00)	121.67 (91.43, 161.61)	155.15 (94.80, 237.88)	0.070
AISI-min	102.67 (72.69, 147.17)	97.63 (68.21, 131.18)	109.89 (77.53, 176.36)	0.043*
AISI-max	194.05 (125.50, 300.92)	167.29 (124.57, 233.04)	224.33 (128.01, 333.06)	0.043*
AISI-mean	139.92 (101.51, 214.03)	130.85 (97.59, 183.96)	174.18 (105.47, 260.90)	0.023*
SII-0	398.15 (301.82, 555.22)	365.98 (293.95, 479.05)	459.95 (313.68, 613.22)	0.005*
SII-1	361.46 (265.48, 519.45)	357.19 (262.90, 487.26)	385.23 (292.05, 594.86)	0.108
SII-2	368.47 (272.32, 474.89)	339.81 (267.05, 438.35)	410.37 (283.16, 550.48)	0.017*
SII-min	302.02 (230.34, 406.84)	267.90 (222.65, 362.69)	344.99 (237.41, 440.09)	0.014*
SII-max	492.20 (356.39, 656.31)	460.73 (354.20, 572.31)	553.57 (369.34, 763.31)	0.010*
SII-mean	388.42 (299.18, 532.19)	359.44 (292.94, 463.24)	451.62 (315.04, 600.33)	0.004*

Continuous variables are presented as mean ± standard deviation for normally distributed data or median (interquartile range) for non-normal data; categorical variables are reported as count (percentage). *$P < 0.05$.

Table S2 Comparison of characteristics between the training and test sets.

Variables	Total (n = 202)	Training set (n = 162)	Test set (n = 40)	*P*-value
Response, n (%)				1.000
Responder	106 (52.48)	85 (52.47)	21 (52.40)	
Non-responder	96 (47.52)	77 (47.53)	19 (47.50)	
Sex, n (%)				0.503
Male	99 (49.01)	77 (47.53)	22 (55.00)	
Female	103 (50.99)	85 (52.47)	18 (45.00)	
Laterality, n (%)				0.549
RE	102 (50.50)	84 (51.85)	18 (45.00)	
LE	100 (49.50)	78 (48.15)	22 (55.00)	
RVO-Type, n (%)				0.816
BRVO	146 (72.28)	116 (71.60)	30 (75.00)	
CRVO	56 (27.72)	46 (28.40)	10 (25.00)	
Anti-VEGF, n (%)				0.092
Ranibizumab	142 (70.30)	115 (70.99)	27 (67.50)	
Conbercept	48 (23.76)	35 (21.60)	13 (32.50)	
Aflibercept	12 (5.94)	12 (7.41)	0 (0.00)	
Diabetes, n (%)				0.223
No	183 (90.59)	149 (91.98)	34 (85.00)	
Yes	19 (9.41)	13 (8.02)	6 (15.00)	
Age	60.50 (52.25, 70.00)	61.50 (53.00, 70.00)	58.00 (52.00, 69.00)	0.470
BCVA-0	0.82 (0.52, 1.23)	0.82 (0.52, 1.23)	0.92 (0.60, 1.07)	0.363
BCVA-1	0.6.00 (0.40, 0.92)	0.60 (0.30, 0.92)	0.70 (0.40, 1.00)	0.190
BCVA-2	0.60 (0.30, 0.92)	0.52 (0.22, 0.82)	0.70 (0.52, 0.92)	0.113
BCVA-min	0.40 (0.22, 0.82)	0.40 (0.22, 0.70)	0.60 (0.38, 0.92)	0.112
BCVA-max	0.92 (0.60, 1.37)	0.92 (0.60, 1.37)	1.00 (0.70, 1.33)	0.380
BCVA-mean	0.64 (0.45, 1.06)	0.64 (0.44, 1.04)	0.73 (0.54, 1.14)	0.217
CMT-0, μm	483.00 (359.00, 633.75)	472.00 (351.00, 633.75)	517.00 (392.75, 623.50)	0.199
CMT-1, μm	284.00 (254.25, 359.00)	287.50 (256.00, 362.00)	274.00 (242.75, 333.50)	0.299
CMT-2, μm	268.00 (243.00, 317.00)	264.00 (241.00, 311.50)	292.50 (255.50, 346.50)	0.055
CMT-min, μm	258.50 (234.50, 289.00)	257.50 (234.00, 289.75)	260.50 (239.25, 285.25)	0.937
CMT-max, μm	508.50 (385.00, 672.50)	498.00 (371.00, 673.00)	578.00 (452.75, 654.00)	0.151
CMT-mean, μm	364.33 (308.50, 456.33)	353.33 (306.83, 448.17)	376.50 (325.92, 474.25)	0.178
N-0, 10^9/L	3.94 (3.18, 4.75)	4.00 (3.19, 4.76)	3.66 (3.16, 4.55)	0.475
N-1, 10^9/L	3.75 (2.98, 4.56)	3.84 (3.06, 4.55)	3.59 (2.88, 4.72)	0.444
N-2, 10^9/L	3.68 (3.09, 4.42)	3.68 (3.06, 4.43)	3.69 (3.20, 4.16)	0.913
N-min, 10^9/L	3.34 (2.68, 3.97)	3.30 (2.69, 3.99)	3.36 (2.62, 3.73)	0.559
N-max, 10^9/L	4.38 (3.66, 5.28)	4.40 (3.68, 5.30)	4.16 (3.62, 5.02)	0.533
N-mean, 10^9/L	3.86 (3.18, 4.54)	3.90 (3.19, 4.54)	3.72 (3.10, 4.38)	0.496
L-0, 10^9/L	1.86 ± 0.52	1.88 ± 0.51	1.78 ± 0.58	0.303

L-1, 10^9/L	1.90 ± 0.53	1.90 ± 0.53	1.90 ± 0.56	0.962
L-2, 10^9/L	1.86 (1.51, 2.30)	1.86 (1.53, 2.29)	1.82 (1.47, 2.32)	0.940
L-min, 10^9/L	1.64 ± 0.44	1.65 ± 0.42	1.60 ± 0.53	0.610
L-max, 10^9/L	2.14 ± 0.54	2.15 ± 0.54	2.12 ± 0.56	0.784
L-mean, 10^9/L	1.89 ± 0.47	1.90 ± 0.46	1.86 ± 0.51	0.724
M-0, 10^9/L	0.36 (0.28, 0.45)	0.36 (0.29, 0.45)	0.34 (0.27, 0.41)	0.297
M-1, 10^9/L	0.37 (0.30, 0.44)	0.37 (0.30, 0.44)	0.37 (0.31, 0.42)	0.955
M-2, 10^9/L	0.38 (0.30, 0.47)	0.37 (0.30, 0.46)	0.41 (0.29, 0.54)	0.205
M-min, 10^9/L	0.32 (0.26, 0.38)	0.32 (0.26, 0.38)	0.32 (0.26, 0.39)	0.771
M-max, 10^9/L	0.42 (0.35, 0.55)	0.42 (0.35, 0.53)	0.41 (0.37, 0.57)	0.556
M-mean, 10^9/L	0.37 (0.31, 0.47)	0.37 (0.31, 0.46)	0.38 (0.31, 0.48)	0.778
P-0, 10^9/L	188.00 (158.25, 220.00)	189.50 (159.25, 222.75)	173.50 (151.25, 201.50)	0.081
P-1, 10^9/L	191.00 (152.25, 221.75)	191.50 (154.25, 225.75)	176.50 (148.75, 210.50)	0.280
P-2, 10^9/L	184.50 (154.25, 222.75)	186.50 (155.00, 223.75)	168.00 (144.50, 222.00)	0.401
P-min, 10^9/L	165.00 (135.00, 192.75)	170.50 (135.00, 198.00)	160.00 (136.25, 178.75)	0.150
P-max, 10^9/L	208.00 (171.00, 239.75)	211.50 (177.00, 241.00)	202.50 (166.25, 224.00)	0.177
P-mean, 10^9/L	188.50 (158.42, 216.58)	191.67 (160.67, 219.25)	180.67 (155.17, 201.33)	0.142
NLR-0	2.09 (1.67, 2.74)	2.09 (1.69, 2.73)	2.11 (1.63, 2.83)	0.816
NLR-1	2.04 (1.58, 2.61)	2.06 (1.58, 2.62)	1.89 (1.61, 2.43)	0.761
NLR-2	1.94 (1.61, 2.51)	1.92 (1.62, 2.48)	2.02 (1.58, 2.81)	0.674
NLR-min	1.75 (1.43, 2.09)	1.74 (1.44, 2.06)	1.80 (1.39, 2.16)	0.951
NLR-max	2.52 (1.93, 3.18)	2.50 (1.93, 3.18)	2.64 (1.96, 3.26)	0.725
NLR-mean	2.16 (1.66, 2.57)	2.15 (1.67, 2.56)	2.23 (1.62, 2.58)	0.789
PLR-0	101.59 (82.00, 123.15)	103.99 (84.32, 122.83)	94.75 (80.11, 128.02)	0.441
PLR-1	100.69 (80.01, 122.32)	103.85 (82.09, 122.09)	94.58 (76.61, 124.79)	0.267
PLR-2	100.62 (83.40, 121.31)	100.62 (83.40, 123.21)	98.65 (83.96, 117.49)	0.486
PLR-min	86.42 (67.96, 101.69)	87.59 (68.62, 102.99)	78.54 (67.42, 94.22)	0.187
PLR-max	117.93 (100.00, 146.07)	117.85 (100.87, 149.62)	118.92 (93.90, 137.55)	0.464
PLR-mean	102.52 (85.86, 122.52)	103.59 (87.16, 124.17)	95.55 (85.37, 115.10)	0.344
LMR-0	4.93 (3.97, 6.40)	4.93 (4.02, 6.29)	4.93 (3.77, 6.83)	0.799
LMR-1	4.96 (4.00, 6.15)	4.92 (4.02, 6.18)	5.26 (3.92, 5.82)	0.792
LMR-2	5.04 (3.92, 6.07)	5.04 (4.06, 6.03)	5.05 (3.50, 6.23)	0.504
LMR-min	4.26 (3.44, 5.13)	4.31 (3.58, 5.20)	3.65 (2.97, 5.09)	0.126
LMR-max	5.92 (4.73, 7.25)	5.92 (4.73, 7.16)	5.89 (4.85, 7.35)	0.959
LMR-mean	5.06 (4.14, 6.05)	5.12 (4.23, 6.05)	5.01 (3.97, 5.93)	0.596
SIRI-0	0.78 (0.54, 1.13)	0.78 (0.56, 1.12)	0.87 (0.46, 1.14)	0.960
SIRI-1	0.76 (0.53, 1.04)	0.78 (0.51, 1.04)	0.69 (0.55, 0.98)	0.431
SIRI-2	0.71 (0.54, 1.00)	0.71 (0.55, 0.96)	0.70 (0.51, 1.32)	0.725
SIRI-min	0.57 (0.44, 0.78)	0.59 (0.44, 0.78)	0.56 (0.40, 0.81)	0.615
SIRI-max	0.99 (0.71, 1.38)	0.99 (0.72, 1.33)	1.09 (0.66, 1.68)	0.433
SIRI-mean	0.79 (0.58, 1.06)	0.79 (0.59, 1.06)	0.82 (0.53, 1.26)	0.727
AISI-0	144.80 (98.81, 220.57)	146.73 (104.25, 222.85)	127.88 (80.35, 202.64)	0.288

AISI-1	132.03 (94.03, 208.81)	133.20 (96.50, 219.29)	103.41 (86.64, 181.57)	0.138
AISI-2	127.25 (93.65, 202.00)	130.21 (95.36, 196.56)	116.42 (84.32, 248.29)	0.792
AISI-min	102.67 (72.69, 147.17)	108.29 (77.12, 152.16)	90.00 (68.13, 115.29)	0.135
AISI-max	194.05 (125.50, 300.92)	194.05 (127.34, 298.79)	191.95 (118.13, 312.45)	0.796
AISI-mean	139.92 (101.51, 214.03)	145.20 (103.21, 214.03)	130.50 (100.12, 214.24)	0.586
SII-0	398.15 (301.82, 555.22)	408.35 (296.72, 558.34)	345.97 (306.38, 505.14)	0.311
SII-1	361.46 (265.48, 519.45)	381.01 (275.72, 526.84)	356.99 (237.85, 446.27)	0.221
SII-2	368.47 (272.32, 474.89)	363.23 (274.36, 477.94)	376.89 (262.06, 453.57)	0.680
SII-min	302.02 (230.34, 406.84)	304.00 (230.34, 413.81)	275.98 (228.97, 360.08)	0.302
SII-max	492.20 (356.39, 656.31)	495.77 (356.51, 662.63)	428.80 (357.21, 615.52)	0.496
SII-mean	388.42 (299.18, 532.19)	408.18 (297.76, 543.70)	356.66 (304.47, 464.81)	0.453

Continuous variables are presented as mean ± standard deviation for normally distributed data or median (interquartile range) for non-normal data; categorical variables are reported as count (percentage).

Table S3 Highly correlated variable pairs in the training set.

Variables 1	Variables 2	ρ	*P*-value
BCVA-0	BCVA-max	0.946	< 0.001
BCVA-1	BCVA-min	0.907	< 0.001
BCVA-1	BCVA-mean	0.911	< 0.001
BCVA-2	BCVA-min	0.923	< 0.001
BCVA-min	BCVA-mean	0.934	< 0.001
BCVA-max	BCVA-mean	0.943	< 0.001
CMT-max	CMT-mean	0.933	< 0.001
N-min	N-mean	0.919	< 0.001
N-max	N-mean	0.942	< 0.001
L-min	L-mean	0.937	< 0.001
L-max	L-mean	0.952	< 0.001
M-max	M-mean	0.942	< 0.001
P-1	P-mean	0.915	< 0.001
P-min	P-mean	0.936	< 0.001
P-max	P-mean	0.965	< 0.001
NLR-max	NLR-mean	0.934	< 0.001
PLR-max	PLR-mean	0.941	< 0.001
LMR-min	LMR-mean	0.921	< 0.001
LMR-max	LMR-mean	0.953	< 0.001
SIRI-min	SIRI-mean	0.908	< 0.001
SIRI-max	SIRI-mean	0.964	< 0.001
AISI-min	AISI-mean	0.932	< 0.001
AISI-max	AISI-mean	0.972	< 0.001
SII-max	SII-mean	0.945	< 0.001

Table S4 Stepwise regression for collinearity-controlled feature preselection.

Variables	Coefficient	Standard Error	t-value	*P*-value
BCVA-0	-0.286	0.106	-2.701	0.008
BCVA-min	0.280	0.123	2.286	0.024
N-max	-0.135	0.057	-2.358	0.020
L-min	0.496	0.227	2.185	0.030
NLR-max	-0.527	0.203	-2.595	0.010
LMR-min	0.191	0.121	1.575	0.118
LMR-max	0.170	0.096	1.767	0.079
SIRI-min	-0.509	0.361	-1.412	0.160
SIRI-max	0.876	0.316	2.775	0.006
AISI-max	-0.007	0.003	-2.658	0.009
SII-max	0.004	0.001	2.987	0.003
CMT-mean	0.001	0.000	4.188	< 0.001
M-mean	-3.676	1.612	-2.281	0.024
NLR-mean	0.565	0.289	1.954	0.053
LMR-mean	-0.422	0.213	-1.984	0.049
AISI-mean	0.012	0.005	2.310	0.022
SII-mean	-0.004	0.002	-2.079	0.039

Table S5 Optimized hyperparameters for each machine learning model selected via five-fold cross-validation and Bayesian optimization.

Model	Parameter	Value
XGBoost	max_depth	5.000
	eta	0.268
	gamma	1.231
	colsample_bytree	0.641
	min_child_weight	4.000
	subsample	0.893
	nrounds	8.000
LightGBM	num_leaves	11.000
	learning_rate	0.056
	max_depth	7.000
	feature_fraction	0.862
	bagging_fraction	0.708
	bagging_freq	3.000
	nrounds	199.000
CatBoost	iterations	104.000
	depth	5.000
	learning_rate	0.100
	l2_leaf_reg	9.019
AdaBoost	n_estimators	136.000
	nu	0.133
	max_depth	3.000
RF	mtry	1.000
	ntree	152.000
	nodesize	6.000
ENLR	alpha	1.000
	lambda	0.000
SVM	C	5.523
	sigma	0.104
KNN	k	5.000
MLP	hidden_layer_sizes	[2]

Table S6 Counterfactual simulations of single and joint interventions on SII-min and NLR-min.

Response	CMT-2	SII-min	NLR-min	SII-gain	NLR-gain	Joint-gain
NonResponder	380.00	514.10	2.10	≤0.00	≤0.00	0.665
	449.00	592.91	1.86	≤0.00	≤0.00	0.649
	246.00	385.00	4.18	≤0.00	0.385	0.597
	216.00	450.28	1.63	0.443	≤0.00	0.586
	246.00	439.48	1.75	0.496	≤0.00	0.527
	264.00	492.40	1.96	0.304	≤0.00	0.515
	268.00	587.98	3.64	0.091	0.289	0.483
	237.00	474.03	1.68	0.392	≤0.00	0.479
	700.00	392.11	2.28	≤0.00	0.888	0.455
	335.00	444.44	2.31	<0.00	0.284	0.387
	241.00	578.95	2.10	0.164	≤0.00	0.345
	283.00	505.64	2.28	≤0.00	0.162	0.325
	346.00	626.78	2.42	0.028	0.311	0.256
	273.00	446.57	2.60	≤0.00	≤0.00	0.251
	262.00	452.87	2.13	0.129	≤0.00	0.236
	294.00	599.29	2.95	0.074	0.146	0.221
	472.00	304.61	2.36	≤0.00	0.402	0.209
	410.00	441.91	2.63	≤0.00	0.054	0.177
	251.00	658.38	2.72	0.488	0.129	0.166
	246.00	320.90	2.41	≤0.00	0.089	0.155
	260.00	475.90	2.96	≤0.00	0.01	0.093
	287.00	527.29	3.71	0.014	0.175	0.092
	360.00	658.38	3.29	0.003	0.02	0.064
	487.00	352.91	2.61	≤0.00	0.035	0.052
	241.00	470.49	2.32	≤0.00	≤0.00	0.035
	407.00	362.00	2.81	≤0.00	0.015	0.031
	304.00	478.70	3.40	≤0.00	0.086	0.021
	342.00	658.38	4.18	<0.001	<0.001	0.004
	641.00	275.43	2.67	≤0.00	0.003	0.002
	727.00	537.24	2.71	≤0.00	0.003	0.001
	641.00	389.87	2.93	≤0.00	<0.001	<0.001
	529.00	547.36	4.18	<0.001	<0.001	<0.001

2 Supplementary Figures

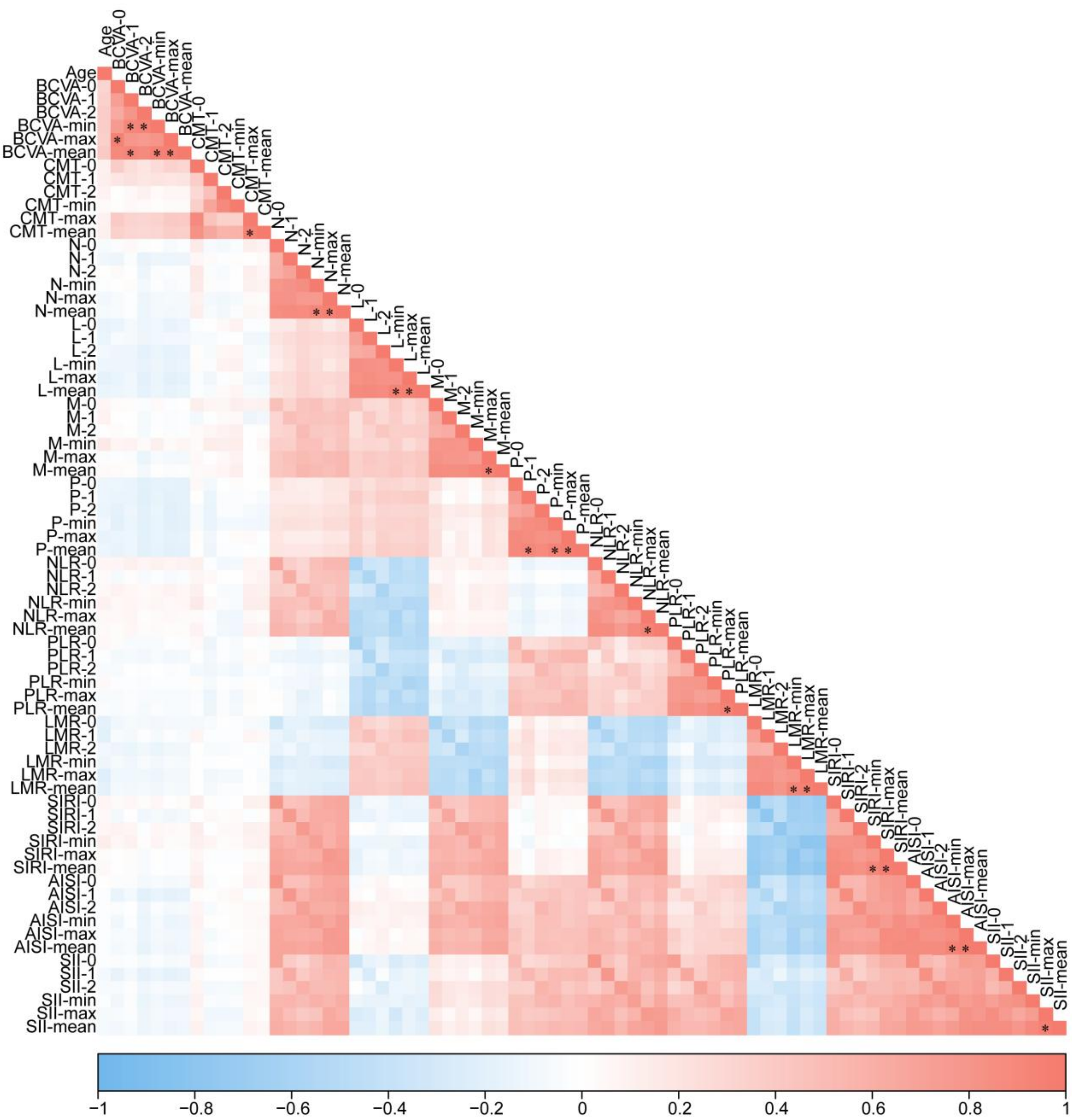

Supplementary Figure 1. Spearman correlation heatmap of numeric variables in the training set. Strong collinearity (|ρ| > 0.90, $P < 0.05$) is marked with asterisks.

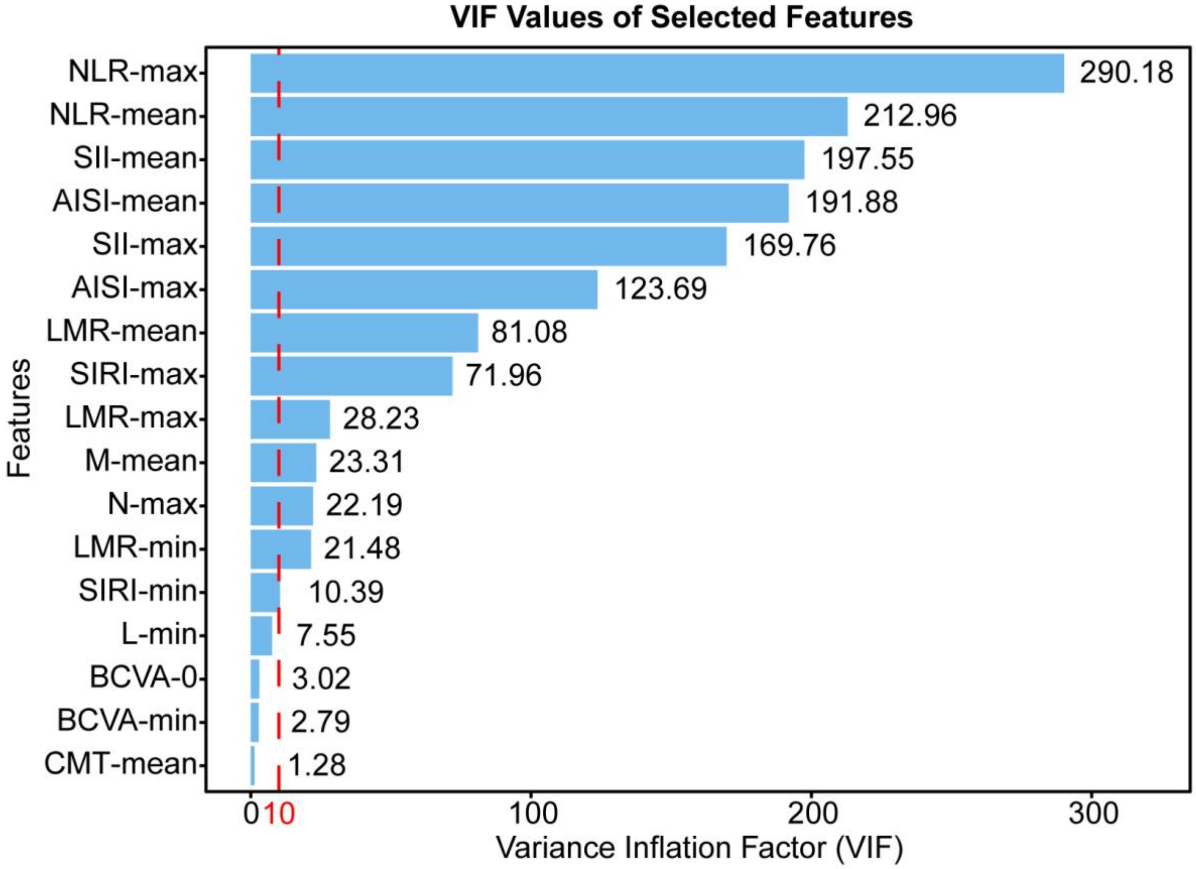

Supplementary Figure 2. VIF analysis of stepwise-selected variables. Bars show VIF values; variables with VIF > 10 (red) were excluded due to multicollinearity.

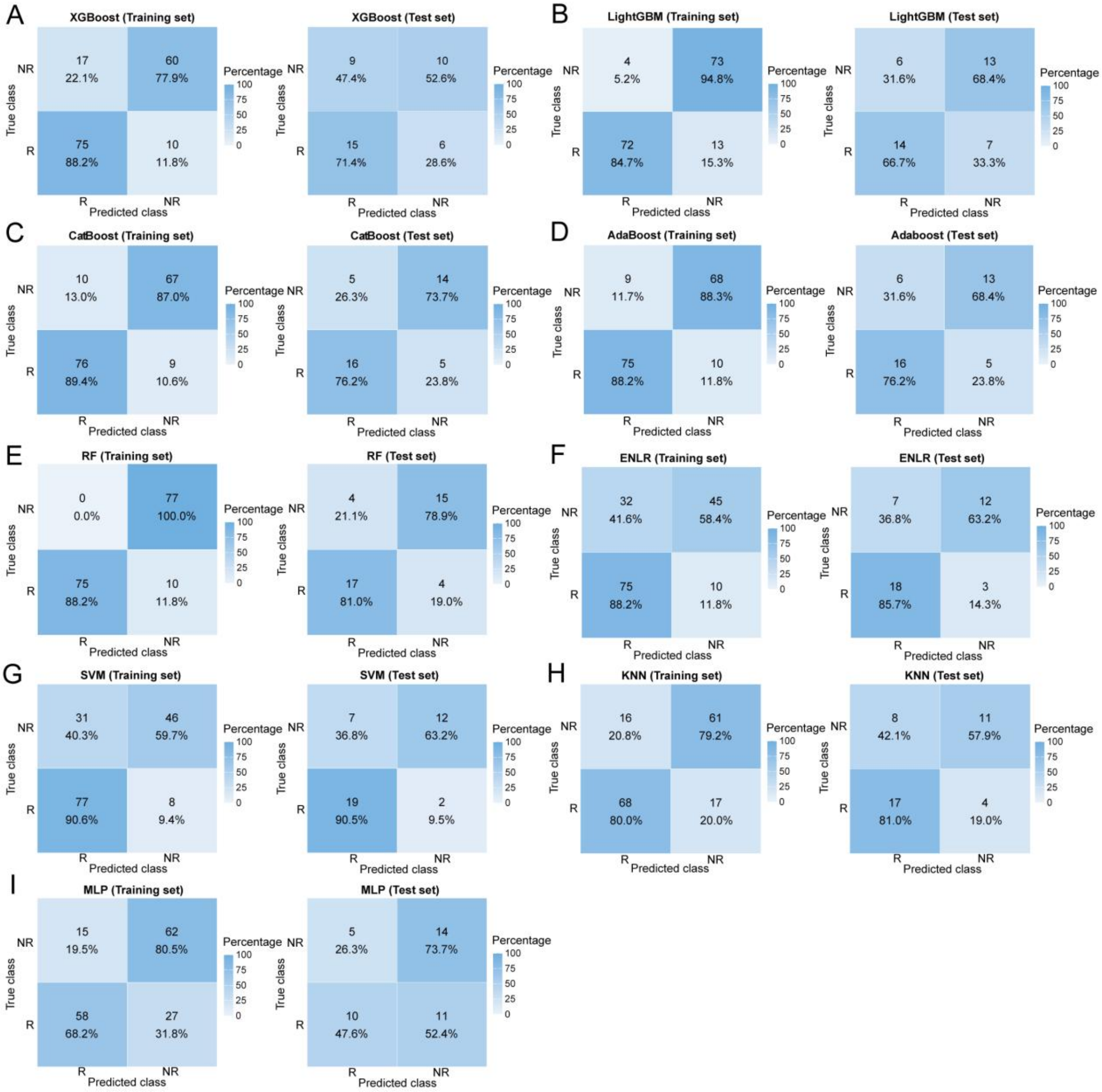

Supplementary Figure 3. Confusion matrices of nine machine learning models on training and test sets. R = response; NR = non-response.

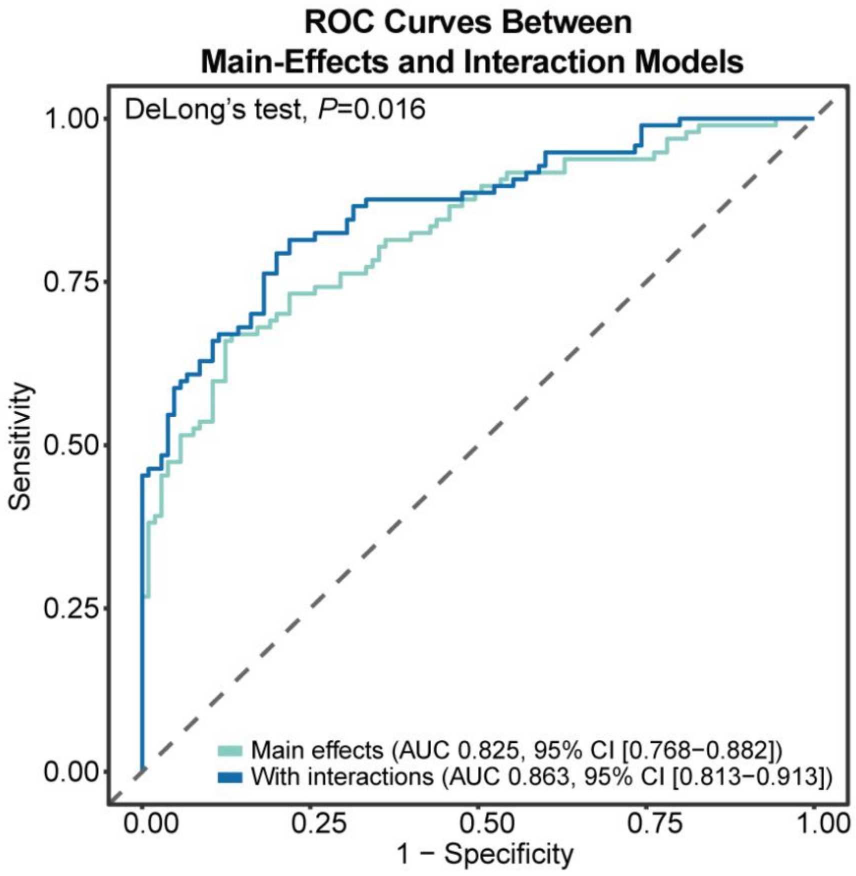

Supplementary Figure 4. ROC curves for the main-effects model and the interaction model. DeLong's test showed a significant difference between the AUCs (P = 0.016).
